# Electrospun Fiber Scaffolds for Engineering Glial Cell Behavior to Promote Neural Regeneration

**DOI:** 10.3390/bioengineering8010004

**Published:** 2020-12-29

**Authors:** Devan L. Puhl, Jessica L. Funnell, Derek W. Nelson, Manoj K. Gottipati, Ryan J. Gilbert

**Affiliations:** 1 Department of Biomedical Engineering, Rensselaer Polytechnic Institute, 110 8th Street, Troy, NY 12180, USA; puhld@rpi.edu (D.L.P.); funnej@rpi.edu (J.L.F.); nelsod4@rpi.edu (D.W.N.); gottim2@rpi.edu (M.K.G.); 2 Center for Biotechnology and Interdisciplinary Studies, Rensselaer Polytechnic Institute, 110 8th Street, Troy, NY 12180, USA; 3 Center for Brain and Spinal Cord Repair, Department of Neuroscience, The Ohio State University, 460 W. 12th Avenue, Columbus, OH 43210, USA

**Keywords:** glia, electrospun fibers, Schwann cells, astrocytes, oligodendrocytes, microglia

## Abstract

Electrospinning is a fabrication technique used to produce nano- or micro- diameter fibers to generate biocompatible, biodegradable scaffolds for tissue engineering applications. Electrospun fiber scaffolds are advantageous for neural regeneration because they mimic the structure of the nervous system extracellular matrix and provide contact guidance for regenerating axons. Glia are non-neuronal regulatory cells that maintain homeostasis in the healthy nervous system and regulate regeneration in the injured nervous system. Electrospun fiber scaffolds offer a wide range of characteristics, such as fiber alignment, diameter, surface nanotopography, and surface chemistry that can be engineered to achieve a desired glial cell response to injury. Further, electrospun fibers can be loaded with drugs, nucleic acids, or proteins to provide the local, sustained release of such therapeutics to alter glial cell phenotype to better support regeneration. This review provides the first comprehensive overview of how electrospun fiber alignment, diameter, surface nanotopography, surface functionalization, and therapeutic delivery affect Schwann cells in the peripheral nervous system and astrocytes, oligodendrocytes, and microglia in the central nervous system both in vitro and in vivo. The information presented can be used to design and optimize electrospun fiber scaffolds to target glial cell response to mitigate nervous system injury and improve regeneration.

## 1. Introduction

Electrospinning is a versatile technique used to generate biocompatible, biodegradable scaffolds for tissue engineering applications. Electrospun polymer fibers are frequently used in regenerative medicine, drug delivery, and in vitro modeling for a variety of biomedical applications, including wound healing, bone regeneration, and nervous system regeneration [1,2,3,4,5,6]. Here, we provide a review of the literature involving electrospun fibers for nervous system regeneration, specifically focusing on the interactions of glial cells with electrospun fibers. Glia are non-neuronal regulatory cells that play a critical role in maintaining homeostasis in the healthy nervous system and regulating regeneration in the injured nervous system. The principal glia discussed here are peripheral nervous system Schwann cells and central nervous system astrocytes, oligodendrocytes, and microglia. We will first describe the general methods used to fabricate electrospun fiber scaffolds for nervous system regeneration applications and how they can be tailored to affect cellular responses. Then, we briefly explain the functions of each of the principal glial cell types in both healthy and pathological states to provide the foundation for how electrospun fiber scaffolds can be designed and optimized to target glial cell response to mitigate nervous system injury, as well as improve regeneration and functional outcomes.

**Figure 1:**
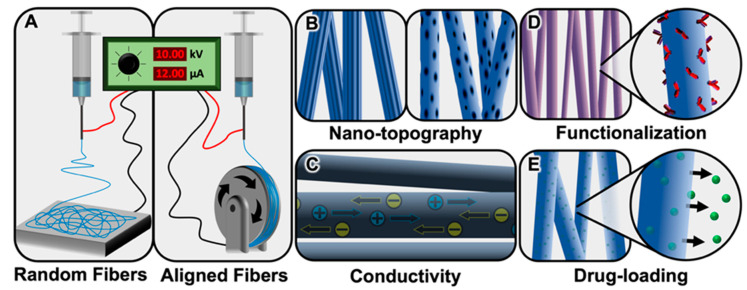
Electrospinning apparatus configurations and examples of electrospun fiber features that affect glial cell behavior. (**A**) Electrospinning apparatus configuration for generating randomly oriented fibers on a flat plate (left) and aligned fibers on a rotating mandrel (right). Electrospinning apparatus parameters and the materials used for electrospinning can be tuned to alter fiber (**B**) nanotopography, (**C**) conductivity, (**D**) functionalization, or (**E**) drug-loading to engineer a desired cell response.

Electrospinning is a fabrication technique used to produce nanometer or micrometer diameter fibers using an electric voltage drop to draw a thin polymer jet from a metal needle and deposit fibers onto a grounded collector [7,8]. The solvent used to dissolve the polymer evaporates as the jet rapidly elongates and whips through the air [9]. The whipping results in the random collection of thin polymer fibers on the collector, usually a metal plate. However, the orientation of the fibers can be controlled by collecting the fibers on a rotating mandrel [10,11] (Figure 1A), an oscillating collection plate [12], or between two parallel plates [13,14]. Studies using electrospun fibers as scaffolds for peripheral nerve or spinal cord regeneration have shown that an aligned fiber arrangement is critical for directionally guiding neuronal regeneration through the injury site, as the aligned fibers mimic the aligned topography of the uninjured anatomy in peripheral nerves and the white matter tracts found in the spinal cord [15,16,17,18,19,20,21,22]. 

The process of electrospinning is versatile in that several parameters can be adjusted to produce fibers with desired properties. Fiber diameter can range from tens of nanometers to tens of micrometers and is modified by altering the concentration of the polymer solution, the dielectric constant and vapor pressure of the solvent, the polymer solution flow rate, and the distance between the needle tip and the collector [17,23]. The nanotopography on the surface of each individual fiber can also vary from smooth to pits, divots, pores, or grooves by incorporating a nonsolvent into the electrospinning solution or varying the humidity of the electrospinning environment [24,25,26] (Figure 1B). Electrospun fiber diameter, alignment, density, and surface nanotopography have all been shown to affect neuronal and glial behavior [24,27,28,29]. Additionally, the unique properties of various materials used to generate electrospun fibers, such as biocompatibility, degradation rate, and conductivity (Figure 1C), can affect cell adhesion, migration, and phenotype [30,31,32]. Fibers can also be further functionalized by incorporating drugs or surface coatings to produce a desirable effect for a given application [1,6,23,33] (Figure 1D,E). In this review, we briefly discuss the role of glia in the nervous system in both healthy and pathological states. We then thoroughly examine the effect of different electrospun fiber properties, namely diameter, alignment, surface nanotopography, conductivity, and functionalization, on glial cell behavior in both in vitro and in vivo environments. 

### Healthy and Pathological Glial Activity

**Figure 2:**
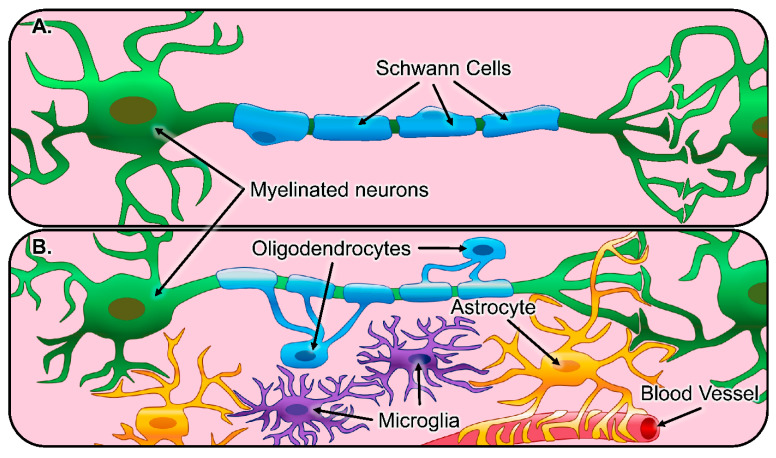
Illustration of glial cells and their function in the healthy nervous system. (**A**) The peripheral nervous system consists of Schwann cells (blue) that myelinate the axons of peripheral neurons (green). (**B**) The central nervous system consists of astrocytes (yellow) that regulate synaptic connections and comprise the blood–brain barrier, oligodendrocytes (blue) that myelinate axons of neurons, and microglia (purple), which act as resident innate immune cells.

In both the peripheral nervous system (PNS) and the central nervous system (CNS), neurons are surrounded by a diverse assortment of regulatory cells collectively called glia. Neuronal interactions with glia are vital for both maintaining a homeostatic balance throughout the neural extracellular matrix (ECM) as well as enabling proper electrophysiological function. The principal glial cell type in the PNS is the Schwann cell [34,35]. In the healthy PNS, Schwann cells myelinate peripheral nerves, increasing the conduction velocity of axonal impulses [36] (depicted in Figure 2A). However, the abundant population of Schwann cells in the PNS serves many purposes outside of their role as the neuron-myelinating cell [37]. This includes enhancing and guiding axon regeneration, calcium signaling to modulate synaptic neurotransmission, and chemokine signaling to recruit nearby cells [38,39]. Following damage to the PNS, mature Schwann cells go through a series of phenotypic changes to support improved regeneration. This process begins as mature Schwann cells dedifferentiate into an immature state and subsequently differentiate into a repair state, characterized by a decrease in the expression of promyelinating factors and an increase in the expression of growth supportive factors. The repair-Schwann cells proceed to migrate out in front of the injured neurons, elongate, and organize into regenerative tracks, known as bands of Büngner, which support and direct axonal regeneration [34,35]. Finally, the Schwann cells sense the presence of the regenerated axons and redifferentiate into their mature state, increasing the expression of promyelinating factors and remyelinating the regenerated nerve fibers [40,41].

The main glial cell types in the CNS are astrocytes, oligodendrocytes, and microglia (depicted in Figure 2B). Astrocytes are the most prevalent glia in the CNS and serve multiple dynamic biological functions, including the modulation of synaptic connections between neurons, continuous sequestering of toxic species, and providing a physical barrier with other cells, known as the blood–brain barrier, between the CNS and all neighboring vasculature [42,43,44]. While both astrocytes and oligodendrocytes display projections from their cell bodies, those from oligodendrocytes specifically construct the myelin sheath around nearby neurons, a concentric wrapping that improves neuron repolarization and conduction speeds. Additionally, recent literature has demonstrated that oligodendrocyte myelination is not only distinct to single groups of neurons but also exhibits control over synaptic activity and can even manipulate information processing [45,46,47]. Microglia are the resident innate immune cells in the CNS, responding to pathogens and injury and clearing dead cells. Microglia also have developmental functions, such as supporting myelination, oligodendrogenesis, neurogenesis, axon fasciculation, and stimulating synaptic formation and maturation via the release of signaling factors [48]. 

Following damage to the CNS, inflammatory cells are recruited to the lesion by extravasation of leukocytes from damaged blood vessels and migration of resident microglia. Microglia and invading macrophages acquire a reactive phenotype and immediately begin removing debris, stabilizing the injury site, and inciting multiple cellular responses via chemokines, including promoting more inflammation [49,50]. Astrocytes also become reactive after injury, with drastic changes in astrocytic gene expression, proliferation, and morphology. Over a period of two weeks, astrocytes become hypertrophic and form a dense scar that protects intact neural networks from further inflammation and damage but also acts as a physical barrier to regenerating axons [51,52]. Additionally, astrocytes and other cells begin synthesizing and depositing various chondroitin sulfate proteoglycans (CSPGs) into the ECM, many of which are neuroinhibitory. Different types of CNS injury have been shown to induce different reactive phenotypes in astrocytes; neuroinflammation resulted in a neurotoxic reactive phenotype, while ischemia resulted in a neurotrophic reactive phenotype [53,54]. Reactive microglia have since been shown to induce a neurotoxic reactive phenotype in astrocytes, where they rapidly kill neurons and mature, differentiated oligodendrocytes [52,55,56,57]. Reactive microglia also produce many cytotoxic components that are responsible for the eradication of local oligodendrocytes [58]. Oligodendrocyte precursor cells (OPCs) respond to this by migrating to the lesion then proliferating and differentiating, which further forms a structurally layered glial scar and helps stabilize dystrophic axons within the hostile lesion environment [59,60]. Overall, peripheral axons regenerate more readily than central nervous system axons, and many experts attribute this difference to the Schwann cell response to injury. A summary of the main glial cells’ response to traumatic injury is listed in Table 1.

Electrospun fiber scaffolds are used to create in vitro culture systems that recapitulate the topographical features of axons in peripheral nerves and white matter tracts in the spinal cord, allowing for increased physiological relevance when studying interactions between glia and neurons in culture [37,45,48]. Additionally, electrospun fibers are readily incorporated into synthetic nerve grafts and assessed using in vivo animal models for their ability to support the infiltration of glia and axonal regeneration across the length of the injury [15,16,61,62,63,64,65,66,67,68,69]. Fibrous scaffolds have repeatably demonstrated their ability to effectively direct the extension of regenerating axons, but their effect on glial activity following injury is not as established. As observed in vivo, glial cells are responsible for both enabling and restricting neuronal regeneration. For this reason, it is important that electrospun fibers and glial response to fibers are studied using in vitro and in vivo models. However, it is important to note that this review compiles findings from in vitro work that utilized primary or immortalized glial cell lines that were isolated or derived primarily from rodents or humans. Previous research shows that immortalized glial cell lines and can exhibit different characteristics compared to their primary cell counterparts [70,71]. Advances in genomics also led to the finding that rodent cells differ from human cells on both molecular and functional levels [72,73]. Thus, the findings presented from each study may not translate amongst the different cell lines utilized in each study. In subsequent sections, we will first review the in vitro use of fibrous scaffolds with each major glial species from the PNS and CNS. Then, we will highlight important in vivo findings that may elucidate certain fiber characteristics capable of promoting neural regeneration. 

## 2. Peripheral Nervous System Glia and Electrospun Fibers 

The PNS regenerates more readily than the CNS, but if injury severity results in a lesion size greater than 1–2 cm in length, then surgical intervention is required to promote regeneration and functional restoration [74,75]. Autografts, in which a donor nerve is isolated from the injured individual and used to bridge the injury, are the current gold standard for peripheral nerve reconstruction. However, the number of appropriately sized nerves are limited, and nerve isolation can lead to donor site morbidity [74,75,76,77,78,79,80]. Allografts, isolated from a cadaver, are a popular alternative to autografts, as they still contain guidance cues from the native ECM architecture. However, cadaveric tissues are limited, processing of the tissue to create the allograft is often time-consuming and labor-intensive, and their use can trigger an immunogenic response after implantation [74,77,78,80,81,82,83]. To overcome these limitations, researchers are investigating the use of synthetic nerve grafts since they can be readily produced and constructed into size-matched structures with reduced immunogenicity [74,79,81,84,85,86]. Still, smooth hollow nerve grafts are inferior to autografts and allografts because they lack the ECM cues and structural support necessary for robust regeneration [21,81,87,88]. Electrospun fibers provide guidance cues that enhance regeneration in the PNS [89,90,91]. Thus, electrospun fibers are frequently incorporated into experimental designs of synthetic nerve grafts to improve their regenerative capacity to levels similar to that of autografts and allografts [15,61,62,63,64,92,93,94]. The improved regenerative capacity of the PNS is often attributed to the presence of Schwann cells, the principal glia of the PNS [34,35]. There are two types of Schwann cells, myelinating and nonmyelinating, and this review will focus on myelinating Schwann cells. Since Schwann cells are so vital to the proper function of the healthy PNS and regeneration of injured peripheral nerves, it is important to understand how the incorporation of electrospun fibers into synthetic nerve grafts affects this key cell type [21,35,74]. The following sections provide a detailed summary of how changing various characteristics of electrospun fiber mats affects Schwann cells in vitro.

### 2.1. Schwann Cell Response to Electrospun Fibers In Vitro

#### 2.1.1. Schwann Cell Response to Fiber Alignment In Vitro

Electrospun fiber orientation is one of the most common parameters studied to optimize electrospun fibers for their use as peripheral nerve grafts. As mentioned previously, aligned electrospun fibers mimic the aligned orientation of the native nervous system ECM and aid in guiding cell migration and axon regeneration across an injury gap. To more quickly repopulate the injury gap with Schwann cells, in the hopes of increasing the rate of regeneration in the PNS, scaffolds can be preseeded with stem cells and progenitor cells in an attempt to have them differentiate towards the Schwann cell lineage. Modifying fiber alignment is known to influence stem and progenitor cell differentiation into various cell lineages, including Schwann cells [95,96,97,98,99,100,101,102,103]. Ren et al. found that aligned electrospun poly(ether sulfone) fibers enhanced the differentiation of neural crest stem cells towards the Schwann cell lineage compared to randomly oriented fiber mats and smooth tissue culture polystyrene surfaces [100]. Another way to quickly repopulate the nerve graft is to preseed the graft with Schwann cells directly or incorporate features that support improved endogenous Schwann cell infiltration of the graft [63,84,85,104]; thus, researchers have investigated the effects of fiber orientation on Schwann cell maturation, adhesion, proliferation, elongation, and migration. Chew et al. showed that aligned electrospun polycaprolactone (PCL) fibers supported the maturation of Schwann cells by stimulating their expression of promyelination markers and downregulating their expression of immature, nonmyelinating markers compared with Schwann cells cultured on randomly oriented fibers and smooth films [105]. A more recent study, conducted by Radhakrishnan et al., corroborated these findings by showing that culture on aligned poly(lactic-co-glycolic acid) (PLGA) fibers promoted maturation of Schwann cells compared to randomly oriented fibers and smooth films. Radhakrishnan et al. also showed that the aligned electrospun fibers improved Schwann cell adhesion at 3, 6, and 12 h postseeding compared to smooth films and randomly oriented fibers [106]. The increased surface area of electrospun fibers compared to flat materials can support greater Schwann cell adhesion, proliferation, and, in turn, improve their ability to populate the graft [107,108,109]. Li et al. observed increased Schwann cell adhesion after 4 h and increased proliferation after 3 and 7 d of culture on aligned electrospun poly(l-lactic acid) (PLLA) nanofiber yarn compared to PLLA films or smooth tissue culture polystyrene [107]. Valmikinathan et al. also found that the increased surface area provided by aligned and randomly oriented PCL electrospun fibers spun onto smooth or porous PCL conduits enhanced Schwann cell adhesion and proliferation. The randomly oriented fibers showed the greatest cell adhesion 1 d postseeding and aligned fibers showed the greatest proliferation 4 d postseeding compared to smooth and porous PCL conduits alone [108]. Similarly, Gnavi et al. observed increased Schwann cell adhesion on randomly oriented GL/PEO_GPTMS (gelatin/polyethylene-oxide/3-Glycidoxypropyl methyldiethoxysilane) nanofibers compared to aligned fibers 3 h postseeding. However, they also found the randomly oriented fibers to support greater Schwann cell proliferation compared to the aligned fibers following 3, 5, and 7 d of cultures [109]. 

In previously mentioned studies, both Chew et al. and Radhakrishnan et al. showed that aligned electrospun fibers improved uniaxial elongation in the direction of fiber orientation [105,106]. This improved elongation can promote the formation of a layer of elongated Schwann cells that structurally mimic the bands of Büngner [105,106,110], which play a pivotal role in guiding regenerating axons directionally across the injury site [34]. Several additional studies have also shown that Schwann cells orient and elongate uniaxially along aligned electrospun fibers [14,15,64,107,108,111,112,113]. For example, Jefferies and Wang designed nerve grafts consisting of several microchannels lined with aligned PCL fibers or randomly oriented PLGA fibers and observed an elongated Schwann cell morphology on the aligned fibers and rounded morphology on the random fibers. Additionally, the graft containing aligned PCL fibers supported the infiltration of Schwann cells into and throughout the graft [14]. Fiber orientation has been shown to affect the direction and rate of Schwann cell migration, [15,63,66,111,114,115], which can control how quickly Schwann cells infiltrate and populate nerve grafts. Schwann cells are known for migrating into the injury gap ahead of axons to support their regeneration into and across the injury gap [116], so it is important to understand how orientation impacts their migration. Wang et al. observed an approximately 4.5-fold increase in average Schwann cell migration distance following culture on aligned poly(propylene carbonate) (PPC) fibers compared to randomly oriented fibers [115]. Similarly, Mukhatyar et al. found that the average Schwann cell migration distance was approximately 4.3-fold greater following culture on aligned poly-acrylonitrile methyl acrylate (PAN-MA) fibers compared to smooth films. The increased migration on the aligned PAN-MA fibers was attributed to fiber orientation as well as the enhanced adsorption of fibronectin, an ECM adhesion molecule [117], from the serum-containing media onto the aligned PAN-MA fibers compared to smooth films. This increased adsorption was thought to be partially due to the increased surface area of the aligned fiber mats compared to the smooth film controls [114]. 

#### 2.1.2. Schwann Cell Response to Fiber Diameter In Vitro

Electrospun fiber diameter is another key characteristic that is modified to optimize electrospun fiber mats for various applications. Several studies have demonstrated that fiber diameter can also play a role in influencing the differentiation of stem and progenitor cells into various cell lineages, including Schwann cells [97,98,100,118,119,120,121]. In a previously mentioned study conducted by Ren et al., it was discovered that 600-nm diameter, as well as 1.6-μm diameter electrospun fibers, in combination with fiber alignment, improved neural crest stem cell differentiation towards the Schwann cell lineage compared to aligned and randomly oriented fiber mats consisting of fibers with a smaller average diameter of 160 nm [100]. Similarly, Xue et al. showed that electrospun fiber mats with an average diameter of 1 μm improved the differentiation efficiency of bone marrow stem cells towards the Schwann cell lineage compared with those that have a smaller average fiber diameter of 500 nm, and fiber alignment played a role in further improving this differentiation [120]. Although the effects of fiber diameter on Schwann cell maturation have yet to be investigated, Cao et al. hypothesized that electrospun fiber diameter would influence Schwann cell maturation because electrospun fibers of nano- and micron-scale diameters mimic ECM protein fibers and axons [122]. Axon diameter, in particular, is known to influence Schwann cell myelination, with a minimum diameter of ~1 μm being required for myelination to occur [123,124]. 

**Figure 3:**
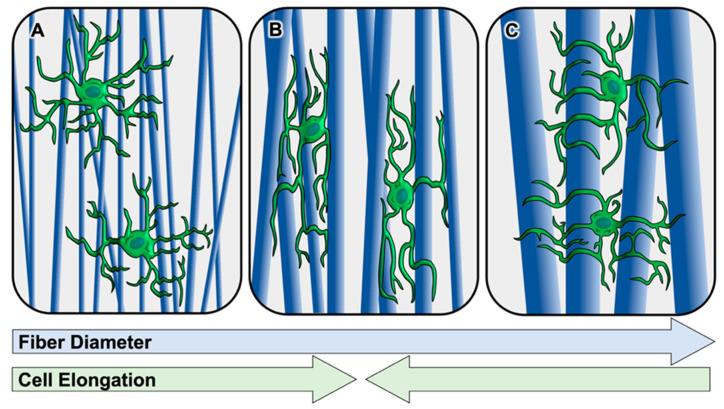
Effect of aligned fiber diameter on glial cell elongation. (**A**) Glial cells spread radially on small diameter fibers. (**B**) Glial cells elongate along the orientation of the aligned fibers on medium diameter fibers. (**C**) Glial cells spread radially on large diameter fibers.

Although fibers of micron-scale diameters seem to improve differentiation towards the Schwann cell lineage and may support the maturation of Schwann cells, Gnavi et al. found that nano-scale gelatin electrospun fibers (300 nm and 600 nm) supported greater Schwann cell adhesion after 24 h and proliferation after 7 d compared to micron-scale fibers (1 μm and 1.3 μm). The nano-scale fibers may have promoted the formation of more focal adhesion sites, thus increasing adhesion and proliferation. Additionally, nano-scale fibers promoted increased spreading of Schwann cells across many fibers, while micron-scale fibers supported greater cell elongation directionally along the orientation of the fibers that the cells came into contact with [125]. This demonstrates that nano-scale fiber diameters can be too small to support uniaxial Schwann cell elongation (depicted in Figure 3A), whereas micron-scale diameter fibers support more robust elongation in the direction of the fibers that the cells contact (depicted in Figure 3B). However, electrospun fiber diameter can also be increased to a size that no longer allows Schwann cells to sense fiber orientation and elongate along them (depicted Figure 3C). Daud et al. found that Schwann cells cultured on aligned electrospun PCL fibers with average diameters of 5 and 8 μm exhibited reduced elongation lengths compared to those cultured on fibers with an average diameter of 1 μm [126]. In addition to affecting Schwann cells’ ability to elongate in the orientation of the fiber, fiber diameter also affects the Schwann cells’ ability to migrate along the fibers. Both Gnavi et al. and Daud et al. observed the greatest Schwann cell migration on fibers with an average diameter of 1–1.3 μm compared to fibers of smaller [125] or larger [126] diameters. These findings agree well with a 2010 study conducted by Wang et al. in which Schwann cells migrated to the greatest lengths following 5 d in culture on aligned PLLA fibers with an average diameter of 1.3 μm compared to those with smaller average diameters of 750 and 300 μm [28]. 

#### 2.1.3. Schwann Cell Response to Fiber Surface Nanotopography In Vitro

In addition to changing larger-scale features of the electrospun fibers, such as fiber orientation and diameter, researchers have investigated the impact of adding smaller nanotopographical features, such as pits, divots, and grooves to fibers on nervous system regeneration [25,127,128,129]. Little has been done to investigate the effect of these nano-scale features on cell differentiation into the Schwann cell lineage or Schwann cell maturation, adhesion, and proliferation. However, the incorporation of these features is shown to increase the roughness of electrospun fibers and, in turn, increase cell adhesion and proliferation [130,131]; thus, we can hypothesize that these features may promote increased adhesion and proliferation of Schwann cells as well. Nano-grooved electrospun fibers, formed through phase separation and the subsequent stretching of the fibers during the electrospinning process, are shown to have beneficial effects on Schwann cells in vitro [132,133]. Huang et al. found that the presence of nanogrooves on electrospun cellulose acetate butyrate fibers improved Schwann cell elongation compared to smooth fibers [132]. Further, Wu et al. observed greater maximum and average Schwann cell migration on nano-grooved electrospun PCL fibers compared to smooth PCL fibers [133].

#### 2.1.4. Schwann Cell Response to Fiber Conductivity In Vitro

Fabricating certain materials into electrospun fibers can help to better recapitulate the native nervous system environment and improve peripheral nerve response to the fibers. For example, conductive materials aid in targeting electrical stimulation to a local region, supporting the transfer of an electric signal to and along neurons to promote improved neurite outgrowth and nervous system regeneration [134,135]; thus, they have been employed in peripheral nerve grafts [134,135,136,137]. In a recently published study, Hu et al. fabricated aligned and randomly oriented PCL/gelatin fibers containing electrically conductive multi-walled carbon nanotubes and investigated their effects on differentiating bone marrow stem cells towards the Schwann cell lineage. They found that aligned control and aligned conductive fibers improve the efficiency of differentiation compared to random control and random conductive fibers, with the aligned conductive fibers promoting the greatest levels of differentiation out of all the fiber groups. In the healthy PNS, Schwann cells typically run along the aligned and electrically conductive axons of peripheral neurons, so the aligned conductive fibers may be more indicative of the native PNS environment and therefore improve differentiation towards the Schwann cell lineage [138]. Biocompatibility can be of concern when incorporating conductive materials into electrospun fiber scaffolds, so it is important to assess their impact on Schwann cell adhesion, elongation, and proliferation to ensure that the material is not causing more harm than good [139,140,141,142,143]. Zhao et al. combined the conductive properties of graphene with highly biocompatible silk fibroin at various concentrations to construct conductive graphene/silk fibroin electrospun fibers. These fibers supported cell survival and Schwann cell proliferation at concentrations of up to 10% graphene. However, higher concentrations of 15% and 20% graphene led to decreased levels of proliferation following 3 days in culture [139]. Zhang et al. coated aligned electrospun PLLA fibers with a layer of conductive graphene-oxide, a common derivative of graphene, and found that these coated conductive fibers were biocompatible, supported the uniaxial elongation of Schwann cells, and improved Schwann cell proliferation following 3 and 6 days in culture compared to control PLLA fibers. This coating led to an increased roughness of the surface, which could be responsible for the increase in Schwann cell elongation and proliferation observed [141].

#### 2.1.5. Schwann Cell Response to Fiber Functionalization In Vitro

Although electrospun fiber mats mimic the structural architecture of the ECM, polymer fibers alone lack the native biochemical cues provided by ECM proteins. However, the functionalization of the surface of electrospun fibers with ECM proteins, such as laminin or fibronectin, or peptides derived from their specific binding motifs, better mimics the native nervous system environment and improves cell response and regenerative outcomes [144]. Since the inclusion of these biochemical cues better mimics the native nervous system environment, they have been shown to improve the differentiation of stem and progenitor cells towards the neural lineage, including Schwann cell lineage [103,120,121,145]. In a previously mentioned study conducted by Xue et al., it was found that, in addition to the use of aligned fibers with a diameter of 1 μm, coating these surfaces with the ECM protein laminin further improved the differentiation of bone marrow stem cells towards the Schwann cell lineage compared with uncoated fiber mats [120]. In a more recent study, Xue et al. used these findings to construct a nerve graft with a tubular honeycomb structure lined with aligned electrospun fibers with an average diameter of 1 μm and coated with laminin. This graft successfully supported the differentiation of the bone marrow stem cells towards the Schwann cell lineage [121]. These ECM proteins and their peptide derivatives facilitate Schwann cell adhesion and proliferation by interacting with adhesion proteins on the cell surface, such as integrins [146,147,148]. Zheng et al. used click chemistry to functionalize aligned and randomly oriented electrospun PCL fibers with the fibronectin-derived peptide GRGDS alone or along with the laminin-derived peptide YIGSR (GRGDS + YIGSR). Both GRGDS and GRGDS + YIGSR functionalized PCL fibers increased Schwann cell adhesion and GRGDS functionalized PCL fibers increased Schwann cell proliferation compared to control PCL fibers. Additionally, aligned functionalized and nonfunctionalized PCL fibers increased Schwann cell elongation compared to all randomly oriented fiber groups [146]. Similarly, Rajabi et al. found that laminin-functionalized electrospun silk fibroin (SF)/poly (ethylene oxide) (PEO) nanofibrous scaffolds improved Schwann cell attachment, growth, and proliferation compared to control fibers and tissue culture polystyrene [147]. The incorporation of these cues can also promote the migration of Schwann cells along the electrospun fibers [148,149,150]. Bockelmann et al. found that aligned electrospun PCL/star-shaped NCO-poly (ethylene glycol)-stat-poly (propylene glycol) (PCL/sPEG) fibers functionalized with GRGDS (PCL/sPEG-GRGDS) increased the rate and distance of Schwann cell migration compared to PCL or PCL/sPEG control fibers [149]. In a more recent study, Chen et al. used a novel approach by functionalizing aligned and randomly oriented PLLA fibers with decellularized peripheral nerve matrix gel. This gel was rich with ECM proteins and growth factors and, when coated onto random and aligned electrospun fibers, increased Schwann cell migration. Further, this decellularized matrix gel also facilitated nerve fiber remyelination by Schwann cells in vitro, indicating that the gel supported the maturation of the Schwann cells [150]. In addition to using native ECM proteins and their peptide derivatives to functionalize electrospun fibers, researchers have employed biochemical cues not native to the PNS ECM to enhance Schwann cell adhesion, proliferation, elongation, and migration [151,152,153]. For example, Feng et al. functionalized the surface of aligned poly (l-lactic acid-co-ε-caprolactone) (PLCL) electrospun fibers with the protein avidin and cultured biotinylated Schwann cells onto them. They observed increased Schwann cell adhesion onto the avidin treated fibers compared to control fibers without any long-term effects on Schwann cell proliferation or elongation [152]. 

#### 2.1.6. Schwann Cell Response to Fiber Therapeutic Delivery In Vitro

In addition to functionalizing the surface of electrospun fibers, therapeutic molecules such as small-molecule drugs, proteins, and nucleic acids (e.g., DNA, miRNA, and siRNA) can be immobilized onto the surface or encapsulated within electrospun fibers to allow for prolonged, local therapeutic delivery [154,155]. Small-molecule drug-loaded electrospun fibers can be utilized to alter Schwann cell activity or affect the Schwann cell maturation state [156,157]. For example, Bhutto et al. fabricated an aligned poly (l-lactic acid-co-caprolactone)/silk fibroin fiber mesh loaded with vitamin B5 via blend electrospinning and observed greater Schwann cell proliferation and dispersion across the entirety of the scaffold compared to unloaded control fibers. This may be attributed to improved scaffold surface wettability with B5 incorporation, making it more conducive to supporting the growth of Schwann cells, or the fact that vitamin B5 has been shown to increase cellular metabolic activity [156]. In another study, Puhl et al. found that the incorporation of the drug fingolimod hydrochloride, known to shift Schwann cells to an immature repair state—into highly aligned PLGA electrospun fibers—increased Schwann cell migration compared with control PLGA fibers. Additionally, the sustained release of fingolimod reduced the Schwann cell expression of mature, promyelinating markers [157]. Following injury or in a disease state, the PNS can be subjected to various forms of stress that can lead to increased Schwann cell death [158]. However, small-molecule drugs can also be incorporated into electrospun fibers to aid in protecting cells from various forms of stress [159]. In a recent study, Zech et al. cultured Schwann cells onto PLGA fibers loaded with the neuroprotective drug, Nimodipine, and found that sustained release of Nimodipine from PLGA fibers significantly reduced Schwann cell death from osmotic and heat stress and, although not significant, slightly reduced Schwann cell death from oxidative stress compared to control PLGA fibers [159]. Loading proteins, such as growth factors, hormones, and enzymes, into electrospun fibers can significantly enhance their regenerative potential in the PNS as well as throughout the body [160,161]. Neurotrophic growth factors, such as nerve growth factor (NGF), neurotrophin-3 (NT-3), brain-derived neurotrophic factor (BDNF), and glial cell line-derived neurotrophic factor (GDNF), are commonly employed in biomaterials for neural repair due to their ability to enhance neuron survival, axonal growth, and Schwann cell migration [162,163,164,165,166]. However, electrospinning neurotrophic factors is costly, and the use of organic solvents and high voltage can diminish their bioactivity [167,168]. Due to these concerns, it is necessary to study alternative and novel means of incorporating neurotrophic factors into biomaterial constructs containing electrospun fibers for neural repair applications [141,143,169,170,171]. Bovine serum albumin (BSA) and heparin are often incorporated to help stabilize neurotrophic factors during biomaterial fabrication [143,160,170,171]. Additionally, neurotrophic factors can be electrospun into coaxial electrospun fibers and reside in a less harsh aqueous core or immobilized to the surface of electrospun fibers via crosslinking or coating [112,143,170,172]. Zhang et al. fabricated aligned coaxial electrospun fibers with an aqueous core, containing NGF stabilized with BSA and a sheath consisting of a blend of conductive polyaniline (PANi) and PLCL/silk fibroin and studied their effects on Schwann cells. Results showed that these NGF-loaded conductive coaxial electrospun fibers supported increased proliferation of Schwann cells and reduced the toxic effects of PANi on Schwann cells compared to non-NGF-containing control fibers [143]. In a more recent study, Zhang et al. fabricated aligned PLLA electrospun fibers coated with heparin/collagen layers encapsulating NGF and found that the heparin/collagen/NGF coated PLLA electrospun fibers improved Schwann cell proliferation and uniaxial elongation compared to uncoated PLLA fibers [112]. 

#### 2.1.7. Summary of Schwann Cell Response to Electrospun Fibers In Vitro

The in vitro studies presented here show the capability of electrospun fiber scaffolds to be fabricated to better mimic the native PNS and enhance differentiation of stem cells towards the Schwann cell lineage, as well as increase Schwann cell adhesion, proliferation, elongation, and migration. Specifically, previous work shows that aligned electrospun fibers with an average diameter of approximately 1 μm are optimal for promoting stem cell differentiation towards the Schwann cell lineage and directional Schwann cell elongation and migration. Schwann cell adhesion and proliferation can be further improved on nano-scale, randomly oriented fibers; however, these fiber characteristics reduce Schwann cell elongation and migration and therefore may reduce regenerative outcomes. To further enhance Schwann cell adhesion and proliferation on aligned micron-scale fibers, the fiber mat can be functionalized with ECM proteins or peptides, or the fibers can be loaded with a therapeutic that enhances Schwann cell interactions with the fibers. These findings are summarized together in Table 2. Due to the success observed in vitro, researchers have used these findings as a basis for fabricating synthetic nerve grafts that support robust peripheral nerve regeneration. In the following section, we will discuss how the electrospun fiber parameters investigated with Schwann cells in vitro effect Schwann cells in vivo.

### 2.2. Schwann Cell Response to Electrospun Fibers In Vivo

#### 2.2.1. Schwann Cell Response to Fiber Alignment In Vivo

Testing the potential of electrospun fiber-containing nerve grafts in vivo is imperative to understanding how Schwann cells respond to the synthetic graft and whether the Schwann cell response leads to improvement of peripheral nerve regeneration. The traumatic injury model most often used to investigate the efficacy of electrospun fibers in the PNS is a sciatic nerve complete transection injury, where a bilateral segment of the sciatic nerve is removed. Preclinical studies often utilize rats as the model organism and bridge an injury gap, typically spanning from 1 cm to critical lengths of upwards of 3 cm, with the synthetic graft implanted within such a gap [173]. Currently, there are nine commercially available FDA-approved peripheral nerve grafts on the market. These grafts mainly consist of a hollow, smooth tube-like structure and are only approved for small injury gaps of 3 cm or less [84]. To increase the efficacy of artificial nerve grafts for critical size peripheral nerve defects (>1 cm in rats; >3 cm in humans), researchers are employing materials with guidance cues, such as those provided by electrospun fibers, to mimic the organization of the native ECM and enhance regeneration [85,86]. Incorporating the topographical cues of electrospun fibers into hollow nerve grafts can enhance Schwann cell infiltration into nerve grafts and support the remyelination of nerve fibers compared to smooth surface, hollow grafts [65,66,111]. Biazer et al. found that nerve grafts lined with randomly oriented poly(3-hydroxybutyrate-co-ε-hydroxy-valerate) electrospun fibers supported the infiltration of Schwann cells and the formation of myelinated nerve fibers, whereas no myelinated fibers were observed in the smooth polymer film control grafts 4 months following implantation into a 30-mm rat sciatic nerve injury model [65]. In another example, Santos et al. fabricated semialigned Polyactive^®^ poly (ethylene oxide terephthalate) and poly(butylene terephthalate) (PEOT/PBT) electrospun fibers with a diameter of 1.44 µm, combining beneficial structural and highly tunable properties of PEOT/PBT into one nerve graft. This Polyactive^®^ graft increased the cross-sectional area of the regenerating nerve as well as the number of axons myelinated by Schwann cells compared to smooth, inert silicon control grafts 4 months following implantation into 10-mm and 15-mm rat sciatic nerve injury models [111]. Although the randomly oriented and aligned fibers each showed improved performance compared to the smooth surface control, the incorporation of aligned electrospun fibers supports increased the migration of Schwann cells and overall infiltration of the graft compared to those that contain randomly oriented fibers in vitro [114,115] and in vivo [63,64,66]. Jia et al. observed enhanced Schwann cell infiltration and, in turn, axonal regeneration across aligned PLCL electrospun fiber-containing constructs compared to random fiber constructs 3 weeks following implantation into a 10-mm sciatic nerve defect [66].

#### 2.2.2. Schwann Cell Response to Fiber Fillers In Vivo

**Figure 4:**
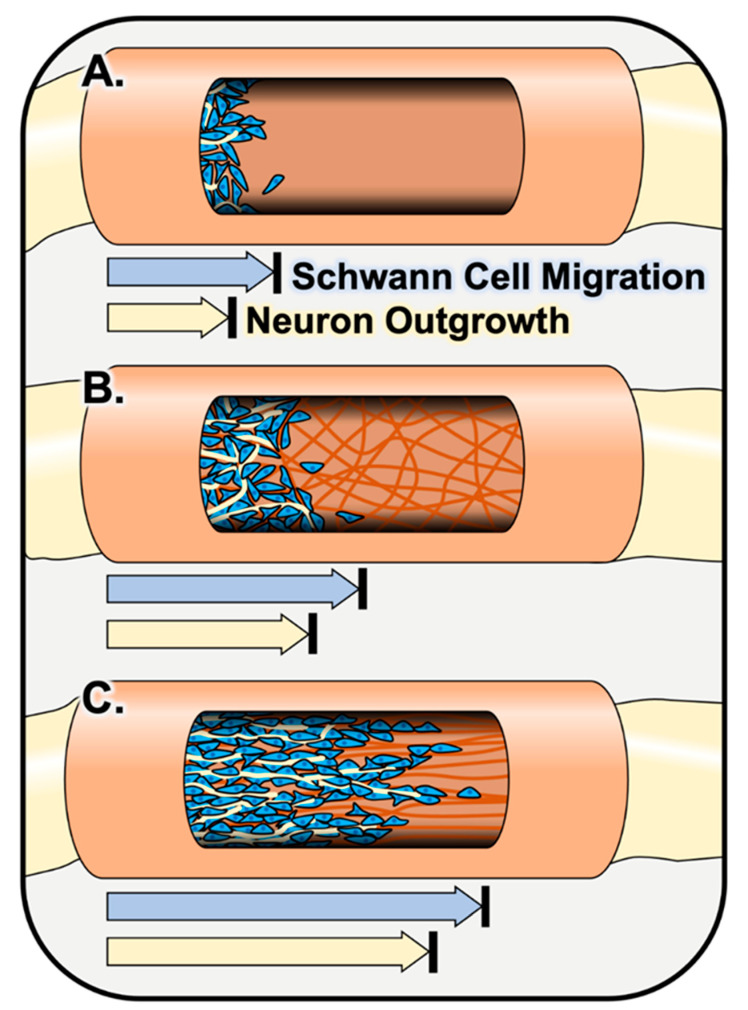
Aligned electrospun fiber conduits promote greater Schwann cell migration and axonal extension over large peripheral nerve gap distances. Synthetic nerve graft bridging the gap between peripheral nerve stumps with either (**A**) a smooth film, (**B**) randomly oriented electrospun fibers, or (**C**) aligned electrospun fibers.

Incorporating a filler consisting of an electrospun fiber matrix throughout the inside of a nerve graft can improve structural support and provides more uniform topographical cues throughout the entirety of the graft. Such fillers have been shown to enhance Schwann cell interactions with nerve grafts compared to hollow nerve graft controls [15,62,63]. Sun et al. incorporated a randomly oriented electrospun fiber sponge, fabricated by electrospinning and subsequent freeze-drying of PLCL/silk fibroin, into a nerve graft and observed increased Schwann cell adhesion and infiltration into and throughout the graft as well as improved remyelination compared to hollow controls 4 and 12 weeks following implantation into a 10-mm rat sciatic nerve injury model [62]. As mentioned previously, the incorporation of electrospun fibers with an aligned orientation can further enhance these regenerative outcomes. Kim et al. observed that aligned electrospun PAN-MA fiber-filled grafts supported Schwann cell infiltration and, in turn, axon regeneration across the entire length of the construct, whereas little to no Schwann cell infiltration nor axon regeneration was observed in random PAN-MA fiber-filled constructs and hollow controls 16 weeks following implantation into a 17-mm rat sciatic nerve defect [63]. Similarly, Du et al. observed improved Schwann cell migration and infiltration and, in turn, axonal regeneration across grafts filled with aligned electrospun fibrin fibers compared to random fibrin fiber and hollow chitosan control grafts 2 weeks following implantation into a 10-mm rat sciatic nerve injury model [15] (depicted in Figure 4). Additionally, immunohistochemical images from both Kim et al. and Du et al. show that Schwann cells on the aligned fiber-containing grafts appear to elongate uniaxially along the aligned fibers, forming tracks mimicking bands of Büngner [15,63]. These observations support in vitro findings previously mentioned in this review [105,106]. 

#### 2.2.3. Schwann Cell Response to Fiber Material Selection In Vivo

As discussed in the in vitro section, materials better suited to mimic the native nervous system environment not only structurally, but also biologically, chemically, and electrically, can be selected to fabricate electrospun fibers in the hopes of supporting improved cell interactions with the electrospun graft and, ultimately, improve regenerative outcomes [15,111]. In the previously mentioned study, Du et al. utilized aligned fibrin electrospun fibers, which possess favorable mechanical properties that mimic those of the native nervous system ECM. In fact, the formation of fibrin cables is an endogenous response following nerve injury; thus, the use of these aligned fibrin fibers can serve to mimic the native repair process both structurally and biochemically to enhance the rate of recovery [15,174]. Additionally, in a study mentioned previously for its in vitro findings, Hu et al. implanted a PCL nerve graft with aligned PCL/gelatin fibers containing electrically conductive multi-walled carbon nanotubes and preseeded with induced, green fluorescent protein (GFP)-labeled bone marrow-derived stem cells into a 10-mm rat sciatic nerve defect model. After 12 weeks, researchers observed differentiation of bone marrow-derived stem cells towards the mature Schwann cell lineage via co-localization of GFP fluorescence and myelin basic protein immunoreactivity. Additionally, the aligned conductive fiber group promoted greater levels of remyelination compared to hollow, smooth PCL control grafts and similar levels of remyelination as the autograft positive control group [138].

#### 2.2.4. Schwann Cell Response to Fiber Functionalization and Therapeutic Delivery In Vivo

Similar to in vitro findings [120,121,146,147,148,149,150], functionalizing electrospun fibers with biochemical cues can significantly enhance their ability to improve peripheral nerve regeneration in vivo [175,176]. Cheong et al. fabricated aligned PLGA electrospun fibers loaded with mussel adhesive proteins fused to bifunctional peptides derived from two different regions of the laminin (IKVAV or YIGSR) protein or the fibronectin protein (GRGDS). The PLGA fibers functionalized with IKVAV performed best in vitro, supporting proliferation, elongation, and differentiation of PC12 neuronal cells and S16 Schwann cells, and this scaffold was selected to implant into a 15-mm rat sciatic nerve injury model. In vivo, the IKVAV functionalized fiber graft supported the repopulation of the graft by neurons and Schwann cells and significantly enhanced remyelination, compared to nonfunctionalized control grafts and the autograft group [175]. In another example, Rao et al. fabricated an aligned electrospun chitosan nanofiber hydrogel functionalized with the growth factor-mimicking peptides, RGI, derived from BDNF, and KLT, derived from vascular endothelial growth factor. In vitro, the functionalized fibers promoted increased Schwann cell proliferation and growth factor secretion. In vivo, the functionalized graft enhanced Schwann cell remyelination of regenerated axons compared to unfunctionalized fiber-containing and hollow graft control groups [176]. Finally, incorporating therapeutic molecules within or on the surface of electrospun fibers can also aid in enhancing the regenerative effects of synthetic nerve grafts in vivo [170,177]. Suzuki et al. found that electrospun fibers loaded with an active form of vitamin B12, known as methylcobalamin, provided local and sustained delivery of the drug, resulting in the enhanced remyelination of axons compared to untreated controls [177]. In another example, Liu et al. fabricated a nerve graft containing coaxial electrospun NGF-loaded fibers consisting of an aqueous BSA/NGF core and PLCL shell and implanted the graft into a 10-mm rat sciatic nerve injury model. In vivo results showed that the NGF-loaded graft enhanced Schwann cell remyelination of regenerated axons and improved the overall regeneration compared to an unloaded graft and unloaded graft plus NGF injection control groups. Furthermore, the NGF-loaded graft performed similarly to the autograft positive control group [170].

#### 2.2.5. Summary of Schwann Cell Response to Electrospun Fibers In Vivo

Due to the challenges and limitations of using autografts and allografts, it is imperative to investigate alternative and more readily available options such as synthetic nerve grafts to promote regeneration and functional recovery following peripheral nerve injury. The in vivo studies reviewed show that electrospun fibers can be incorporated into synthetic nerve grafts to enhance their regenerative potential compared to commercially available synthetic grafts. Further, these studies reveal various parameters and properties of electrospun fibers that can be tuned to enhance Schwann cell infiltration of the graft, remyelination of regenerated axons, and ultimately, regenerative outcomes comparable to that seen with autografts, the current gold standard for peripheral nerve reconstruction. For example, synthetic grafts filled with aligned electrospun fibers improve Schwann cell infiltration throughout the length of the graft compared to hollow grafts or those filled with randomly oriented fibers. Additionally, micron-scale fiber diameter supports Schwann cell remyelination as this diameter mimics the threshold axon diameter required for Schwann cell myelination in the native PNS. Finally, the fibers within the graft can be functionalized or loaded with therapeutics to improve Schwann cell interactions with and response to the graft. These findings, summarized in Table 3, will provide a foundation for future construction of synthetic nerve grafts that aim to enhance regenerative outcomes to levels beyond that of autografts.

## 3. Central Nervous System

Although synthetic nerve grafts containing electrospun fibers have been readily investigated in preclinical in vivo PNS injury models, testing in the CNS has been limited due to the challenges of translating these materials into preclinical CNS injury applications. These challenges arise from the increased complexity and reduced regenerative potential of the CNS compared to that of the PNS [178]. Following PNS injury, Schwann cells differentiate into a phenotype that promotes regeneration [34,35], whereas CNS glial cells take on a reactive and often growth-inhibitory phenotype after CNS injury [56,179]. This reactive phenotype leads to the production of growth-inhibitory factors and the formation of the glial scar, which serves as a physical and chemical barrier to protect the CNS from further damage but also blocks the regeneration of CNS neurons through the injury site [137,180]. Effective treatments for CNS injury must aim to (1) find a balance between reducing this reactive glial phenotype while still protecting the CNS tissue from further damage and (2) stimulate the regeneration of CNS neurons across the injury gap. Electrospun fibers are thought to be a viable treatment option, since they mimic the native nervous system ECM architecture and support and guide cell elongation, migration, and regeneration in a directional manner [17,18]. Additionally, fibers can be designed to deliver therapeutics that modulate cell reactivity, protect cells from postinjury stress, and stimulate neuron regeneration [33,181,182]. Due to the myriad of factors contributing to the lack of regeneration in the CNS, it is important to understand the effects of electrospun fibers on the individual CNS glial cell types. In the following sections, we will discuss astrocyte, oligodendrocyte, and microglia responses to various characteristics of electrospun fibers in vitro. 

### 3.1. Astrocyte Response to Electrospun Fibers In Vitro

As highlighted in the introduction, following CNS injury, astrocytes at the injury site become reactive and ultimately lead to the formation of a glial scar, which acts as a chemical and physical barrier for the regenerating axons. Therefore, targeting and alleviating detrimental characteristics of the astrocytic response has been thought to improve functional benefits after CNS injury. Hence, researchers have studied the use of electrospun fibers to target the astrocytic response, and the expression of glial fibrillary acidic protein (GFAP) has primarily been used as a readout of astrocytic reactivity [183,184,185,186]. Liu et al. showed that astrocytes decrease GFAP expression when cultured on aligned and randomly oriented electrospun collagen fibers, compared to flat collagen-coated glass coverslips [187]. Similar results were also observed when astrocytes were plated on randomly oriented cellulose acetate electrospun fibers [188] and PCL electrospun fibers [189] compared to flat surface controls. Conversely, a recent study by Gottipati et al. further characterized astrocytic response to electrospun fibers by studying the expression of various reactive astrocyte phenotypic markers; they showed that the astrocytes cultured on aligned PLLA fibers display a more neurotoxic reactive phenotype compared to PLLA films [190]. However, it is difficult to directly compare these studies because the PLLA fibers used in this study were larger (micron-scale) in diameter than those used in previous studies (nano-scale). 

Electrospun fibers also support astrocytic expression levels similar to those observed on the in vivo level. Lau et al. found that astrocytes cultured on aligned and randomly oriented PCL electrospun fibers had fewer actin stress fibers and reduced GFAP expression compared to the flat surface controls. Gene expression analysis also showed an upregulation in the genes involved in cell motility and pathfinding along with an increase in the expression of genes that promote neuronal survival including BDNF and glutamate transporter-1 (GLT-1) [191]. Zuidema et al. also observed an increase in the expression of GLT-1 when astrocytes were cultured on randomly oriented or aligned PLLA electrospun fibers compared to flat PLLA film controls, and this increase functionally translated to an increase in the uptake of extracellular glutamate by these astrocytes [29]. A recent study by Zhao et al. using electrospun composite poly(hydroxybutyrate-cohydroxyvalerate), PLLA, and collagen nanofibrous scaffolds also showed an increase in astrocytic expression of neuroprotective genes, such as GLT-1, along with a decrease in the expression of GFAP and the neuroinhibitory CSPGs neurocan and phosphacan compared to astrocytes cultured on flat tissue culture polystyrene [192].

In addition to gene expression, several studies have investigated the ability of electrospun fibers to modulate the morphological features of astrocytes in culture. Like in the PNS, electrospun fibers support directional growth and elongation of CNS glia. Early work showed that polydioxone electrospun fibers supported astrocyte growth along the orientation of the fiber matrix that they were cultured on [193]. Similarly, astrocytes have also been observed growing along the length of electrospun fibers made up of PCL [194,195], PLLA [29,196], collagen [187], silk [197], poly (methyl methacrylate) (PMMA) [198] and PLGA [159]. In a previously mentioned study, Zuidema et al. observed that aligned PLLA electrospun fibers supported greater uniaxial elongation and the migration of astrocytes in the orientation of the fibers compared with randomly oriented PLLA fibers and flat PLLA films [29]. In a more recent study, Zuidema et al. created a topographic transitional boundary between aligned, anisotropic PLLA electrospun fibers and a smooth, isotropic PLLA film and observed that astrocytes seeded on these scaffolds show a shift in their orientation at the anisotropic-to-isotropic fiber/film transition boundary, emphasizing the efficacy of the electrospun fibers in promoting directional elongation of astrocytes [199]. 

#### 3.1.1. Astrocyte Response to Fiber Diameter and Surface Nanotopography

Recent work by Johnson et al. showed that astrocytes react differently to the physical properties of electrospun fibers, such as surface nanotopography and fiber diameter. In the first study, electrospun PLLA fibers were engineered with smooth, pitted, or divoted surface nanotopography and cortical or spinal cord primary rat astrocytes were cultured on these surfaces. The results showed that cortical astrocytes were significantly shorter and broader on the pitted and divoted fibers compared to those on smooth fibers, while spinal cord astrocytes showed no significant changes in their surface features implying that astrocytes from unique anatomical locations respond differently to the presence of nanotopography. Additionally, pitted and divoted fibers restricted spinal cord astrocyte-mediated neurite outgrowth, while smooth fibers enhanced this effect [25]. As a follow-up, the authors assessed the effect of fiber diameter on astrocytes by seeding astrocytes onto aligned PLLA nanofibers with two distinct diameters (808 and 386 nm). The astrocytes on the large-diameter fibers showed significantly increased elongation compared to those on the small-diameter fibers. The authors also note that the astrocytes extending along larger diameter fibers were better equipped to support long neurite outgrowth, and neurite outgrowth along these astrocytes was less branched than outgrowth along astrocytes cultured on small diameter fibers [27].

#### 3.1.2. Astrocyte Response to Fiber Functionalization and Therapeutic Delivery

As mentioned previously, the surface of electrospun fibers can be functionalized to better mimic the native nervous system environment and improve cell response to electrospun fibers. Puschmann et al. coated polyurethane electrospun fibers with poly-l-ornithine and laminin and observed that the functionalized fibers showed a significant decrease in the astrocytic expression of GFAP as well as increased astrocyte adhesion and viability compared to the uncoated fiber controls. An in-depth analysis of astrocytes on these functionalized fibers also showed a significant reduction in the expression of nestin, synemin, vimentin, and heat shock 70, compared to functionalized flat glass controls, suggesting that the functionalized fibers reduce astrocytic cellular stress in culture. They also showed a difference in the expression of genes involved in biochemical pathways that regulate cell proliferation, cell shape determination, and cell motility and note that the functionalized 3D electrospun fiber mat allows astrocytes to maintain some important features that they exhibit in vivo, but that those features are lost in flat, 2D culture environments [200]. 

In addition to the physical interaction of astrocytes with the electrospun fibers, these scaffolds have also been loaded with various biomolecules to modulate astrocytic properties in vitro. Schaub and Gilbert fabricated PLLA electrospun fibers loaded with the antimetabolite 6-aminonicotinamide and found that these drug-releasing fibers attenuate astrocytic metabolic activity in culture compared to unloaded PLLA fiber controls [196]. Similarly, Kim et al. observed that electrospun PCL fibers loaded with the blue-green microalgae Spirulina alleviated the metabolic activity of cultured astrocytes compared to unloaded PCL control fibers [195]. As in the PNS, CNS glia are subjected to various forms of stress following CNS injury. Zech et al., previously mentioned in the in vitro Schwann cell section, fabricated PLGA electrospun fiber mats containing the neuroprotective drug Nimodipine and found that the Nimodipine fiber mats significantly reduced cell death in in vitro cell models of oxidative, osmotic, and heat-induced cell stress in immortalized as well as primary astrocytes [159]. 

#### 3.1.3. Electrospun Fibers for Generating In Vitro Models to Study Astrocyte Behavior 

Finally, electrospun fiber scaffolds have also been used by several groups to create in vitro models of the blood–brain barrier (BBB) [201,202,203,204]. Bischel et al. created an in vitro BBB model using electrospun gelatin biopapers and cocultured human endothelial cells and astrocytes on these biopapers. They showed that the gelatin biopapers had improved transendothelial electrical resistance, decreased permeability, and permitted a smaller separation between cells compared to the standard polyethylene terephthalate (PET) inserts [204]. In a follow-up study, the authors investigated the expression of 27 genes involved in BBB permeability, cellular function, and endothelial junctions at different time points and showed that there was an increase in the expression of the transcripts involved in endothelial junction formation, including TJP2 and CDH5, in the gelatin biopaper model compared to PET inserts [205]. One study has used electrospun fibers to create an in vitro model of spinal cord injury (SCI) by connecting 3D organotypic spinal cord slice arrays to PLLA nanofiber meshes and assessing cellular growth induced by these nanofibers. The aligned fibers promoted directed growth of astrocytes and the neurons were found associated with the oriented astrocytes [206]. 

#### 3.1.4. Summary of Astrocyte Response to Electrospun Fibers In Vitro

The in vitro studies presented here show the capability of 3D electrospun fiber scaffolds to decrease astrocytic reactivity and better recapitulate the in vivo astrocyte phenotype in vitro compared to 2D flat surface controls. Smooth, aligned electrospun fibers with an average diameter of approximately 1 µm increase astrocyte elongation and migration, improving astrocyte-mediated neurite outgrowth compared to pitted/divoted aligned fibers and randomly oriented fibers. Electrospun fiber scaffolds can be improved further by coating them with ECM proteins and/or incorporating drugs to reduce astrocyte stress and reactivity to enhance regenerative outcomes. 

### 3.2. Oligodendroglia Response to Electrospun Fibers In Vitro

After CNS injury, oligodendrocyte populations are vital in remyelinating regenerating axons and improving their conduction velocities, a crucial component of restoring sensory and motor function [59]; thus, it is important to understand how various characteristics of electrospun fibers affect the ability of oligodendrocytes to remyelinate postinjury. Although the effects of electrospun fiber orientation have been readily investigated with other glia, such as Schwann cells and astrocytes, only one study has investigated the effect of fiber orientation on OPCs. Diao et al. demonstrated that randomly oriented PCL electrospun fibers significantly improved OPC differentiation and maturation compared to aligned fibers. Further genomic analysis showed that randomly oriented fibers reduced oligodendroglia expression of differentiation suppressors, which may be attributed to their improved levels of differentiation [207]. 

#### 3.2.1. Oligodendroglia Response to Fiber Diameter In Vitro

Electrospun fiber diameter is a key parameter that has been investigated for its effect on cell differentiation to various lineages, including its influence on neural stem and progenitor cell differentiation towards oligodendrocytic lineage [98,208]. Christopherson et al. first showed that neural stem cells favored this lineage when cultured on poly (ether sulfone) fibers with an average diameter near 0.3 µm, whereas larger diameters favored neuronal lineages [98]. However, this neural stem cell differentiation was induced by the addition of retinoic acid and therefore the study did not delineate the fiber topography’s influence on initiating neural stem cell differentiation. Conversely, Nisbet et al. showed that electrospun PCL fibers alone, even at a larger fiber diameter of 0.75 µm, favored neural stem cell differentiation towards an oligodendrocytic lineage, as opposed to an astrocytic or neuronal lineage [208]. 

Fiber diameter has also been shown to influence oligodendrocyte morphology and myelinating behavior. Lee et al. showed that OPC and oligodendrocyte ensheathment favored polystyrene fibers with diameters greater than 0.4 µm and increased as diameter increased. Coating the smaller fibers with cell adhesive molecules did not improve myelin wrapping, indicating that this threshold may be solely biophysical [209]. Bechler et al. further investigated myelinating behavior between spinal cord and cortical oligodendrocytes cultured on PLLA electrospun fibers [210]. While differentiation potential and ensheathment did not differ between these populations, the lengths of sheaths formed by spinal cord oligodendrocytes were significantly longer than those formed by cortical oligodendrocytes. It was also shown that both populations followed the above trend in which larger diameter fibers produced longer myelin sheaths [210]. 

Electrospun fibers are often used as a neuron-free myelination model system because the fiber diameters are on the same scale as axons. In fact, recent research has primarily used fibers to study the cellular biology of CNS myelination and evaluate the efficacy of therapeutics targeting oligodendroglia. As examples, Fu et al. used PLLA fibers to investigate the unique microtubule biology in oligodendrocytes, while Weightman et al. demonstrated that OPCs rely on the presence of astrocytes to survive and elongate on a 3D fibrous scaffold [211,212]. Others have used electrospun fibers to investigate the oligodendroglial pathobiology of CNS diseases, such as multiple sclerosis, perinatal brain injury, Pelizaeus–Merzbacher disease, and Alexander disease [213,214,215,216]. Additionally, these fibrous scaffolds have been used to screen for potential therapeutics that target underlying molecular mechanisms relating to myelination in CNS disease. Using electrospun fiber model systems, various molecules have already been screened and shown to reduce OPC stress-related death, improve oligodendrocyte maturation, and reverse demyelinating processes [215,217,218,219]. For this reason, electrospun fibers will continue to be used as they provide a facile and reproducible myelinating assay [220]. Additionally, machine learning has already shown promise in providing quick, accurate, and multi-parametric results from these in vitro assays [221]. 

#### 3.2.2. Oligodendroglia Response to Fiber Functionalization and Therapeutic Delivery In Vitro

As mentioned previously, materials that better mimic the native nervous system electrically and biologically can be incorporated onto electrospun fiber scaffolds to improve cell response. For example, Shah et al. coated PCL fibers with the conductive material graphene oxide and showed that this further promoted neural stem cell differentiation towards the oligodendrocyte lineage compared to PCL fibers and graphene oxide alone [222]. Additionally, a study by Li et al. showed that the addition of cell-adhesive gelatin to PCL fibers with diameters above 0.8 µm further improved oligodendrocyte myelination compared to control PCL fibers of similar diameters [223]. Similarly, in a previously mentioned study, Bechler et al. showed that PLLA electrospun fibers functionalized with laminin improved spinal cord-derived oligodendrocyte myelination, however, this was not observed for cortical-derived oligodendrocytes [210].

While electrospun fibers alone have been shown to modulate oligodendrocyte myelination to some extent and have found a unique niche as a model system for myelination assays, recent publications have also highlighted their ability to deliver therapeutics that further enhance the myelinating capabilities of oligodendrocytes. Specifically, one group has heavily investigated the use of electrospun PCL fibers for the local delivery of microRNAs (miRNA) 219 and 338. Initially, Diao et al. showed how miRNA immobilized to the surface of PCL fibers with a 3,4-dihydroxy-l-phenylalanin (DOPA) coating reduced the expression of genes that inhibit OPC differentiation, and therefore promoted oligodendrocyte differentiation and maturation [224]. A follow-up study further investigated the effect of varying fiber diameter and orientation in addition to the miRNA coating. In the absence of miRNA, large diameter and randomly oriented fibers enhanced OPC differentiation and maturation. However, when fibers were coated with the miRNA, the opposite was true in which aligned fibers yielded greater maturation than random fibers, and smaller diameter fibers enhanced OPC differentiation [207]. A study by Ong et al. further advanced this platform in which the electrospun fibers were suspended off the stiff coverslip between two small PCL blocks. Using this modified platform, it was shown that the miRNA-coated fibers not only improved OPC differentiation and maturation but also significantly improved myelination around the fibers, providing an optimized neuron myelination model system [225].

#### 3.2.3. Summary of Oligodendroglia Response to Electrospun Fibers In Vitro

The in vitro studies presented here show how electrospun fibers can impact oligodendroglia differentiation, maturation, and myelination. Randomly oriented electrospun fibers are more beneficial for promoting OPC differentiation, and fiber diameters greater than 0.4 µm improve oligodendrocyte maturation and myelination. Electrospun fiber scaffolds can also be functionalized or loaded with drugs and nucleic acids to further enhance oligodendrocyte maturation and myelination for promoting axonal remyelination after injury. However, the use of fiber scaffolds as an axon-free model system has drawn the most attention. Using such a model can either assist in identifying the basic biological roles of oligodendroglia or can provide a facile therapeutic screening assay. Nevertheless, electrospun fibers also provide many tunable physical parameters for targeting myelination as a therapeutic intervention. While little has been done to investigate fibers as drug depots for myelinating therapeutics, coaxial fibers may provide a unique venue to dually guide axon outgrowth via a bio-functionalized shell, as well as improve myelination via a drug-loaded core. Additionally, nanotopography has yet to be investigated as a tunable parameter for influencing oligodendroglia. Overall, electrospun fibers will continue to provide a unique substrate for neuron-free assays and will require more investigation to be used as a therapeutic intervention for CNS injury. 

### 3.3. Microglia Response to Electrospun Fibers In Vitro

Microglia play a major role during the acute response after injury by clearing debris, modulating inflammatory response, and influencing neurogenesis [49,50,226]; thus, it is important to understand how electrospun fiber characteristics modulate this response. Pires et al. first investigated microglial morphology in response to electrospun fibers. In this study, randomly oriented poly (trimethylene carbonate-co-ε-caprolactone) electrospun fibers were shown to influence an elongated cell morphology compared to the rounded morphology of microglia observed on flat film controls. While microglia cultured on fibers did have an initial increase in proinflammatory (TNFα) cytokine release, the subsequent culture of astrocytes with microglia-conditioned media did not result in astrogliosis, meaning that microglial activation was not significant. Additional morphological analysis showed increased cellular complexity and multinucleation in microglia cultured on films. While this morphology cannot directly presage microglia reactivity, the authors indicate that these differences may result from a reduced activation of microglia on films [227]. Venkateswarlu et al. found that aligned PLGA electrospun fibers supported increased microglia elongation along the orientation of the fibers compared to randomly oriented fibers. While randomly oriented fibers showed more frequent and intense calcium influxes, aligned fibers facilitated a synchronous calcium response, indicating coordination between seeded microglia [228]. This is significant since coordinated glial calcium signaling mirrors the normal physiological function of glia; astrocytes use calcium signaling cascades to modulate synaptic connectivity while microglia rely on calcium waves to locate dying or nonfunctional neurons after injury [229,230].

Unlike astrocytes and oligodendroglia, little has been done to elucidate microglial response to fibrous topography. While these abovementioned studies do provide evidence of basic morphological response to topography, the correlation between fibrous scaffolds and microglial proliferation, migration, and activation remains unclear. Since microglia play a major role during the acute response after injury, understanding this glia-fiber interface and its related biological role can influence the direction of fibrous scaffold interventions for neural tissue engineering. For this purpose, research will surely reveal significant findings regarding electrospun fibers and microglia in the coming years. While additional literature does exist for functionally similar macrophages [127,231,232,233,234,235,236], they go outside the scope of this review as macrophages are not native to the CNS and have been shown to present significantly different and distinct roles after neural injury [237,238,239,240,241].

Though there are fewer studies investigating the effect of electrospun fibers on CNS glia compared to PNS glia, the in vitro studies presented here demonstrate that electrospun fibers enhance elongation, migration, and alter the phenotype of astrocytes, microglia, and oligodendroglia. The CNS glial cell response to various electrospun fiber features is summarized in Table 4. Due to the promising responses to fibers from CNS glia observed in vitro, researchers have explored the fabrication of fiber scaffolds for implantation after CNS injury. In the following section, we will discuss how electrospun fiber scaffolds affect glial cell behavior after injury in vivo.

### 3.4. CNS Glia Response to Electrospun Fibers In Vivo

Testing the efficacy of electrospun fibers in vivo is crucial to understanding how the different glial cell types respond in tandem to the scaffold and how their responses affect the microenvironment and ultimately the regeneration of neurons. Models of traumatic injury used to investigate the efficacy of electrospun fibers in the CNS are most often a type of SCI. Models of SCI include (1) a contusion injury, where a weight is dropped on the spinal cord causing compression, (2) a complete transection injury, where a bilateral segment of the spinal cord is removed, and (3) a hemisection injury, where a unilateral segment of the spinal cord is removed. Fewer studies have examined electrospun fiber scaffolds for brain injury, where injury is induced from a controlled stab or removal of a small area of tissue. The type of injury is important to consider when designing electrospun fiber scaffolds because the native architecture of the spinal cord is different from the brain and the native cells will respond differently to the scaffold. Additionally, the 3D structure of the scaffold is important to consider when moving from in vitro to in vivo; a contusion SCI may need a wafer or thin layer of fibers, while a complete transection may need a dense cylindrical conduit. Nisbet et al. showed that neurites extended into randomly oriented PCL fiber scaffolds implanted in a stab wound lesion in the caudate putamen in the rat brain but did not extend when the fibers were aligned [242]. Conversely, Álvarez et al. showed that aligned poly-L/DL lactic acid 70/30 fibers loaded with l-lactate transplanted into the somatosensory cortex in the brain promoted the formation of new blood vessels and the infiltration of astrocytes, OPCs, and neurons into the scaffold, while no infiltration was observed into randomly oriented fiber scaffolds. In this study, the fiber scaffolds mimicked the 3D organization (aligned electrospun fibers) and supportive function (l-lactate release) of embryonic radial glia, the principal NSCs that generate neurons and glia [69]. Aligned electrospun fibers are also advantageous in the spinal cord because they mimic the aligned orientation of the white matter tracts [16,17,33,187]; a study by Hurtado et al. pioneered the use of aligned electrospun fiber conduits to increase axonal regeneration after a T9-T10 complete transection SCI compared to smooth films and randomly oriented fiber conduits. Astrocytes migrated into the PLLA fiber scaffolds to a similar extent as the axons extended into the scaffold, as though playing a supportive role to the neurons. Migration was decreased in randomly oriented fiber conduits and almost nonexistent in smooth film conduits, mimicking the levels of axonal extension [16]. Conversely, Liu et al. showed no astrocyte infiltration into aligned or randomly oriented collagen fiber scaffolds implanted in a C3 hemisection SCI, though this may be due to the suppressive nature of the collagen fiber scaffolds, which were shown to reduce astrocyte proliferation in vitro. Microglia and macrophages infiltrated the aligned and randomly oriented fiber scaffolds in equal numbers 10 d after implantation, but fewer remained in the aligned fiber scaffolds compared to randomly oriented fiber scaffolds at 30 d [187]. 

The material used to create the electrospun fibers is an important parameter to consider when designing for implantation into the CNS. Chen et al. showed that aligned photocrosslinked gelatin methacryloyl (GelMA) electrospun fiber scaffolds implanted into T9 hemisection SCI facilitate the migration of NSCs and induces their differentiation into neurons compared to aligned gelatin fibers. There was also reduced GFAP and CSPG staining with aligned GelMA fibers compared to aligned gelatin fibers, indicating that neurotoxic astrocyte reactivity was diminished with this material [68]. In another study by Zhao et al., various blends of poly(hydroxybutyrate-cohydroxyvalerate) (PHBV), PLLA, and collagen were used to generate randomly oriented fiber scaffolds implanted into a T10 hemisection SCI. As mentioned previously in the in vitro section, astrocytes cultured on these fibers increased expression of GLT-1 and decreased expression of GFAP and CSPGs. In vivo, PHBV/PLLA/collagen scaffolds diminished astrocyte accumulation at the lesion site compared to PHBV/PLLA fiber scaffolds and smooth scaffold controls. The composition of the fibers even altered the outcomes on the functional recovery level; animals with PHBV/PLLA: collagen (50:50) blend fiber scaffolds had significantly higher motor recovery scores compared to PHBV/PLLA:collagen (70:30) blend fiber scaffolds [192]. 

#### 3.4.1. CNS Glia Response to Fiber Density In Vivo

In addition to the type of material used and the alignment of the fibers, fiber density (number of fibers per area) in the scaffold has been shown to affect glial cell response in vivo. Cnops et al. compared high- and low-density poly (caprolactone-co-ethyl ethylene phosphate) (PCLEEP) aligned fiber scaffolds for enhancing regeneration after a T9-T10 complete transection SCI. High-density fiber scaffolds showed a significant reduction in glial scarring compared to low-density aligned fiber scaffolds [67]. Another study injected PLLA electrospun fibers embedded at high and low densities within an agarose/methylcellulose hydrogel into the striatum of a rat brain. Astrocytes infiltrated the scaffold in the high-density fiber group but created a barrier similar to the glial scar around the lesion in the low-density fiber group [243]. These studies indicate that a higher fiber density is advantageous for promoting astrocyte infiltration into the implanted scaffold and reducing glial scarring. 

#### 3.4.2. CNS Glia Response to Therapeutic Delivery from Electrospun Fibers In Vivo

Due to the limited intrinsic regenerative capacity in the CNS and the complexity of the injury cascades that occur after trauma, researchers are developing hybrid approaches to target multiple aspects of the regenerative process, such as inflammation, glial cell reactivity, and neurite outgrowth. Electrospun fibers can be functionalized or loaded with drugs to target multiple aspects with one scaffold [244,245,246,247]. Aligned poly (trimethylene carbonate-co-ε-caprolatone) fibers loaded with ibuprofen and implanted into a T8 hemisection SCI helped minimize inflammation and promoted astrocyte and anti-inflammatory microglia infiltration into the lesion [227]. Randomly oriented PPC fibers loaded with dibutyryl cyclic adenosine monophosphate (dbcAMP) implanted into a T8 hemisection SCI reduced glial scar area compared to PPC fibers alone. Additionally, motor function scores improved at 2, 3, and 4 weeks after injury with dbcAMP-loaded fiber scaffolds compared to PPC fibers alone [246].

Loading larger and more sensitive molecules such as proteins and enzymes into electrospun fibers can be difficult because the harsh organic solvents and high voltage used in the electrospinning process may diminish the bioactivity of the proteins [33]. To preserve the bioactivity of chondroitinase ABC (ChABC), an enzyme that abates the inhibitory effects of CSPGs on axonal regeneration, Ni et al. encapsulated ChABC in chitosan microspheres and then loaded the microspheres into PPC electrospun fibers [248]. The sustained delivery of ChABC from scaffolds implanted into a T8 hemisection SCI decreased glial scarring and CSPG production compared to the fiber scaffolds alone [248]. Colello et al. used alginate beads to encapsulate both ChABC and NGF for loading into PDS electrospun fibers. After transplanting the scaffolds into a T9-T10 complete transection SCI, astrocytes and myelinating oligodendrocytes surrounding regenerated axons were found within the implant [181]. 

More recent studies have investigated combinatorial approaches to treating SCI: aligned fiber scaffolds for guiding axonal extension combined with growth factor, nucleic acid, and enzyme delivery to promote regeneration [182,249,250]. To assess the translatability of electrospun fiber conduits as an SCI treatment, Gelain et al. investigated implanting the scaffolds at a chronic time point; approximately 10 electrospun fiber tubes of 2–3 mm in length were implanted into a cavity surrounded by gliotic scar tissue 4 weeks after a T8 contusion SCI. The tubes were filled with a self-assembling peptide hydrogel loaded with a cocktail of growth factors: BDNF, ciliary neurotrophic factor, vascular endothelial growth factor, and ChABC. Astrocytes and microglia were found between and around the guidance channels, while myelinated axons were found within the electrospun fiber tubes. The scaffold alone was enough to improve motor function scores at 24 weeks after scaffold implantation, as the addition of the hydrogel and growth factor cocktail did not further improve motor function at the same time point [250]. Coaxial electrospun fibers can protect the bioactivity of proteins from the harsh environment during electrospinning by incorporating them into the core solution [251,252,253,254]. Xi et al. fabricated aligned NGF+HA/PLA core/shell fibers with IL-4 pDNA-loaded liposomes grafted to the fiber surface to target the acute inflammation stage as well as promote neural regeneration in later stages after SCI. Implanted scaffolds reduced inflammation, glial scarring, and improved motor function 4+ weeks after a T9 hemisection SCI compared to control scaffolds with no IL-4 pDNA-releasing liposomes [249]. Another study by Milbreta et al. aimed to promote axonal growth and remyelination by generating scaffolds with aligned PCLEEP fibers embedded in a collagen hydrogel loaded with miRNA-219, miRNA-338, and NT-3 implanted in a C5 hemisection SCI. The delivery of miRNA-219 and miRNA-338 preserved the number of oligodendroglial lineage cells around the injury site, promoted OPC differentiation, and enhanced myelin formation [182]. This is currently one of the only studies that utilizes electrospun fibers to target oligodendrocytes after CNS injury in vivo. 

#### 3.4.3. Suggestions for Future Work Investigating CNS Glia Response to Electrospun Fibers

Many of the studies described here are preliminary investigations into the glial response to electrospun fiber implants, where the main parameters assessed are infiltration and scarring. The main findings of CNS glia response to fiber implants are summarized in Table 5. Unlike for peripheral nerve injury, there are no commercially available synthetic grafts to promote regeneration after CNS injury. This is most likely because it is difficult to implement fibrous scaffolds into common CNS injury geometries and because of the complexity involved in implanting scaffolds into the CNS without causing further damage to surrounding tissue. Therefore, there is more preclinical research utilizing electrospun fibers as an in vitro modelling tool to study fundamental glial cell behavior. It is currently unknown how different electrospun fiber features modulate astrocyte and microglial reactivity or oligodendrocyte myelination behavior in vivo. More investigation is needed to further characterize the CNS glial cell response to electrospun fiber scaffolds so that fiber parameters can be optimized for promoting neural regeneration, as well as how to optimize scaffold implantation for improving functional outcomes. 

## 4. Conclusions

Electrospun fiber scaffolds mimic the native ECM in the nervous system and provide contact guidance for regenerating neurons. The major glia in the PNS and CNS are affected by several electrospun fiber characteristics both in vitro and in vivo. The complex pathophysiology of nervous system injury requires therapies that target multiple aspects of the repair and regeneration process for various durations. Electrospun fiber scaffolds offer a wide range of design parameters that can be tailored to achieve this; fiber diameter, alignment, density, biocompatibility, surface nanotopography, and surface chemistry can be tuned to elicit a desired cell response. Further, electrospun fibers can be loaded with drugs, nucleic acids, and growth factors to provide the local and sustained release of therapeutics to alter the glial cell phenotype and create a regenerative environment. This review highlights the progress that has been made in developing electrospun fiber scaffolds to promote Schwann cell migration across large injury gap distances in the PNS and reduce glial scarring after injury in the CNS. The multitude of in vitro studies presented here provides some insight as to how glial cells are affected by electrospun fibers, but it is important to note that glial cell response to electrospun fiber characteristics in vitro will not always translate to glial cell response in vivo. Further, glial cell response to electrospun fibers in an in vivo rodent model will not always translate to what may be observed at a clinical level in humans. Therefore, more work is needed to (1) understand how electrospun fibers affect glia in vivo, (2) develop hybrid scaffolds that target inflammation, neurons, and glia to improve regeneration and functional recovery after nervous system injury, and (3) determine the efficacy of hybrid electrospun fiber scaffolds in human clinical studies. 

## Figures and Tables

**Table 1:** Summary of glial cell response to traumatic injury.

Schwann Cells	Astrocytes	Oligodendroglia	Microglia
Reduce production of myelinating factors and demyelinate from axons Dedifferentiate into repair state Increase migration and production of neurotrophic factors Redifferentiate into mature myelinating state and remyelinate regenerated axons	Proliferate and migrate to injury site Produce neuroinhibitory molecules Generate glial scar around lesion	Immediately die from excitotoxicity Demyelinate damaged and nearby axons Oligodendrocyte precursor cells proliferate and migrate to lesion to stabilize dystrophic axons	Produce proinflammatory signals Remove cellular debris Activate nearby glia

**Table 2:** Summary of Schwann cell response to various electrospun fiber properties in vitro.

Cell Response to Fiber:	Alignment	Diameter	Surface Nanotopography	Conductivity	Functionalization	Therapeutic Delivery
Schwann cells	Aligned improve stem and progenitor cell differentiation towards Schwann cell lineage [100,120,138]. Aligned improve Schwann cell maturation [105,106], elongation [14,15,64,105,106,107,108,111,112,113,141], and migration [114,115]. Random improve Schwann cell adhesion [108,109].	Diameters of ~600 nm–1.6 µm improve stem and progenitor cell differentiation towards Schwann cell lineage [100,120]. Diameters of ~1–1.3 µm improve Schwann cell elongation and migration [28,125,126]. Diameters <600 nm improve Schwann cell adhesion [125]. Diameters ≥5 µm reduce Schwann cell elongation [126].	Improve Schwann cell elongation [132] and migration [133].	Support stem cell differentiation towards the Schwann cell lineage [138]. Can be toxic at high concentrations [139].	Improve stem cell differentiation towards the Schwann cell lineage [120,121]. Improve Schwann cell adhesion/proliferation [146,147,148,152], migration [148,149,150], and maturation [150].	Improve Schwann cell proliferation [112,143,156] and elongation [112], reduce Schwann cell maturation [157], and protects Schwann cells against stress and toxins [143,159].

**Table 3:** Summary of Schwann cell response to various electrospun fiber properties in vivo.

Cell Response to Fiber:	Alignment	Filler	Material Selection	Functionalization	Therapeutic Delivery
Schwann cells	Aligned improve Schwann cell infiltration of nerve grafts [63,64,66] and uniaxial elongation [15,63].	Filling hollow nerve grafts with electrospun fibers improves Schwann cell adhesion to grafts [62], uniform infiltration of Schwann cells throughout grafts [15,62,63], and remyelination of regenerated axons [62].	Fibers fabricated from natural biopolymers improve Schwann cell response to nerve grafts [62]. Conductive fibers improve stem cell differentiation towards the Schwann cell lineage following graft implantation and improve remyelination of regenerated axons [138].	Improve Schwann cell infiltration of nerve grafts [175] and remyelination of regenerated axons [175,176].	Improve Schwann cell remyelination of regenerated axons [170,177].

**Table 4:** Summary of central nervous system glial cell response to various electrospun fiber properties in vitro.

Cell Response to Fiber:	Alignment	Diameter	Surface Nanotopography	Conductivity	Functionalization	Therapeutic Delivery
Astrocytes	Aligned enhance astrocyte migration and elongation [29].	Larger fibers (808 vs. 386 nm) enhance astrocyte regenerative properties [27].	Pitted and divoted fibers broaden astrocyte morphology and restrict regenerative properties. Smooth fibers enhance astrocyte regenerative properties [25].	Unknown	Enhance astrocyte viability and adhesion. Decrease astrocyte reactivity [200].	Decrease astrocyte metabolic activity [189,190]. Increase astrocyte survival after insult [159].
Oligodendrocytes	Random enhance OPC differentiation and maturation [207]	>0.4 µm induces myelination [209]. >0.5 µm improves OPC maturation and myelination [209,210].	Unknown	Unknown	Promote neural stem cell differentiation towards oligodendrocyte lineage [222]. Improve oligodendrocyte myelination [204,217].	Enhance OPC differentiation and maturation [176,201,218,219].
Microglia	Aligned promote microglial elongation and yield coordinated responses [221,222].	Unknown	Unknown	Unknown	Unknown	Unknown

**Table 5:** Summary of central nervous system glia response to various electrospun fiber properties in vivo.

Cell Response to Fiber:	Alignment	Density	Material Selection	Therapeutic Delivery
Astrocytes	Aligned increase astrocyte infiltration into the scaffold [16,69].	High-density fiber scaffolds reduce glial scarring [67,237]. High-density fiber scaffolds increase astrocyte infiltration into the scaffold [243].	Decrease astrocyte reactivity and accumulation at the lesion [68,186].	Increase astrocyte infiltration into the scaffold [175,221,244]. Reduce glial scarring [240,242,243,244].
Oligodendrocytes	Aligned increase oligodendrocyte precursor cell (OPC) infiltration into the scaffold [69].	Unknown	Unknown	Enhance OPC differentiation and maturation [182]. Increase oligodendrocyte infiltration into the scaffold [175,176].
Microglia	Aligned promote microglial infiltration into the scaffold [187].	Unknown	Unknown	Decrease inflammation [221,243].

## Data Availability

No new data were created or analyzed in this study. Data sharing is not applicable to this article.
